# The association between patellar alignment on magnetic resonance imaging and radiographic manifestations of knee osteoarthritis

**DOI:** 10.1186/ar2138

**Published:** 2007-03-07

**Authors:** Leonid Kalichman, Yuqing Zhang, Jingbo Niu, Joyce Goggins, Daniel Gale, Yanyan Zhu, David T Felson, David J Hunter

**Affiliations:** 1 Boston University School of Medicine, Clinical Epidemiology Research and Training Unit, 650 Albany Street Suite X200, Boston, MA 02118, USA

## Abstract

The aim of our study was to evaluate the association between patellar alignment by using magnetic resonance imaging images and radiographic manifestations of patello-femoral osteoarthritis (OA). Subjects were recruited to participate in a natural history study of symptomatic knee OA. We examined the relation of patellar alignment in the sagittal plane (patellar length ratio (PLR)) and the transverse plane (sulcus angle (SA), lateral patellar tilt angle (LPTA), and bisect offset (BO)) to radiographic features of patello-femoral OA, namely joint space narrowing and patellar osteophytes, using a proportional odds logistic regression model while adjusting for age, sex, and bone mass index (BMI). The study sample consisted of 126 males (average age 68.0 years, BMI 31.2) and 87 females (average age 64.7 years, BMI 31.6), 75% of whom had tibiofemoral OA (a Kellgren-Lawrence score of 2 or more). PLR showed a statistically significant association with joint space narrowing and osteophytosis in the lateral compartment. SA showed significant association with medial joint space narrowing and with lateral and medial patellar osteophytosis. LPTA and BO showed significant association with both radiographic indices of the lateral compartment. Clear linear trends were found in association between PLR, LPTA and BO, and with outcomes associated with lateral patello-femoral OA. SA, LPTA, and BO showed linear trends of association with medial joint space narrowing. Results of our study clearly suggest the association between indices of patellar alignment and such features of patello-femoral OA as osteophytosis and joint space narrowing. Additional studies will be required to establish the normal and abnormal ranges of patellar alignment indices and their longitudinal relation to patello-femoral OA.

## Introduction

Osteoarthritis (OA) is a major public health problem because of its high prevalence, costs, and levels of pain and disability. The prevalence of knee OA makes this disease the single greatest cause of chronic disability in community-dwelling adults in the United States [1,2]. Patellae that are located centrally in the trochlear groove and not malaligned are thought to be less likely to develop OA [3-5]. Patellar malalignment can cause excess stress on the articular surfaces of the patello-femoral (PF) joints and can potentially be a reason for degenerative changes in the knee [6-8].

Most studies of patellar malalignment use plain X-ray evaluations of the knee in the lateral plane and skyline view [6,9-12]. Various methods have been proposed to evaluate patellar malalignment using radiographs: first, in the lateral plane, by evaluation of the relationship between patellar height and patellar ligament length [13,14]; and second, on the skyline view, by evaluation of the trochlear sulcus angle (SA) and depth [15], by evaluation of the lateral PF angle [6,16], the lateral patellar tilt angle (LPTA) [17], and the bisect offset (BO) of the patella [18], and by evaluation of congruence angle [17].

Very few studies have evaluated PF alignment by magnetic resonance imaging (MRI) [19-21]. Muellner and colleagues [19] performed measurements analogous to those used in X-ray evaluation with MRI images obtained with knees flexed to 20° and 45°. Knee flexion allows the evaluation of PF relations when the patella is located in opposition to the femoral trochanteric sulcus. However, in common clinical practice MRI of the knees is usually obtained in the supine position, with fully extended knees. Multiplanar MRI acquisitions permit the assessment of alignment in both the axial and sagittal planes. Therefore in the present study we evaluated patellar alignment on MRI images of extended knees.

Radiography is currently the most widely used method to assess damage in OA [22]. This technique permits the measurement of joint space narrowing and osteophytes, among other features. Regulatory requirements for the development of disease-modifying drugs in OA still consider the measurement of joint space narrowing on plain X-rays to be the appropriate primary endpoint for demonstration of efficacy [23,24]. In this study we used X-ray-evaluated indices of knee OA in medial and lateral PF joints that evaluate such features as joint space narrowing and patellar osteophyte development.

The aim of our study was to evaluate the association between PF alignment (using standard MRI images of extended knees) and radiographic manifestations of PF OA. Our hypothesis was that increasing patellar malalignment on MRI would be positively associated with PF radiographic changes (the presence of joint space narrowing and osteophytes). Factors associated with structural alteration in the PF joint are not as well characterized as in the tibiofemoral joint. This study sought to assess patellar alignment indices that may be selectively associated with the PF joint structural changes.

## Materials and methods

### Study design

The study was designed as a cross-sectional observational study.

### Sample

Subjects were recruited to participate in a natural history study of symptomatic knee OA, called the Boston Osteoarthritis of the Knee Study (BOKS). Subjects in this study are a subset of subjects whose recruitment has been described in detail elsewhere [25]. In brief, subjects were recruited from two prospective studies of the quality of life of veterans (one of men and one of women), from clinics at the Veterans Administration Boston Health Care System and from advertisements in local newspapers. Potential participants were asked two questions: 'Do you have pain, aching or stiffness in one or both knees on most days?' and 'Has a doctor ever told you that you have knee arthritis?' For subjects who answered positively to both questions, we conducted a follow-up interview in which we asked about other types of arthritis that could cause knee symptoms. If no other forms of arthritis were identified, the individual was eligible for recruitment. To determine whether subjects had radiographic OA, they underwent a series of knee radiographs (see below under 'Radiographic evaluation'). If the subject had a definite osteophyte on any view in the symptomatic knee, they were eligible for the study. By having frequent knee symptoms and radiographic OA, all subjects met American College of Rheumatology criteria for symptomatic knee OA [26]. For the natural history study, we enrolled subjects who were interested in participating and who could walk with or without a cane. The examinations were approved by the Boston University Medical Center and the Veterans Administration Boston Healthcare System Institutional Review Boards. Each subject's written consent was obtained in accordance with the Declaration of Helsinki.

### MRI evaluations

All studies were performed with a Signa 1.5T MRI system (General Electric Corp., Milwaukee, WI, USA) using a phased-array knee coil. A positioning device was used to ensure uniformity between patients. The imaging protocol included sagittal spin-echo proton density-weighted and T2-weighted images (repetition time (TR) 2,200 ms; time to echo (TE) 20/80 ms) with a slice thickness of 3 mm, a 1 mm interslice gap, one excitation, a field of view (FOV) of 11 to 12 cm, and a matrix of 256 pixels × 192 pixels; and coronal and axial spin-echo fat-suppressed proton density-weighted and T2-weighted images (TR 2,200 ms; TE 20/80 ms) with a slice thickness of 3 mm, a 1 mm interslice gap, one excitation, and with the same FOV and matrix. The 213 MRIs from BOKS were digitally archived.

### Patellar alignment evaluation

**Figure 1:**
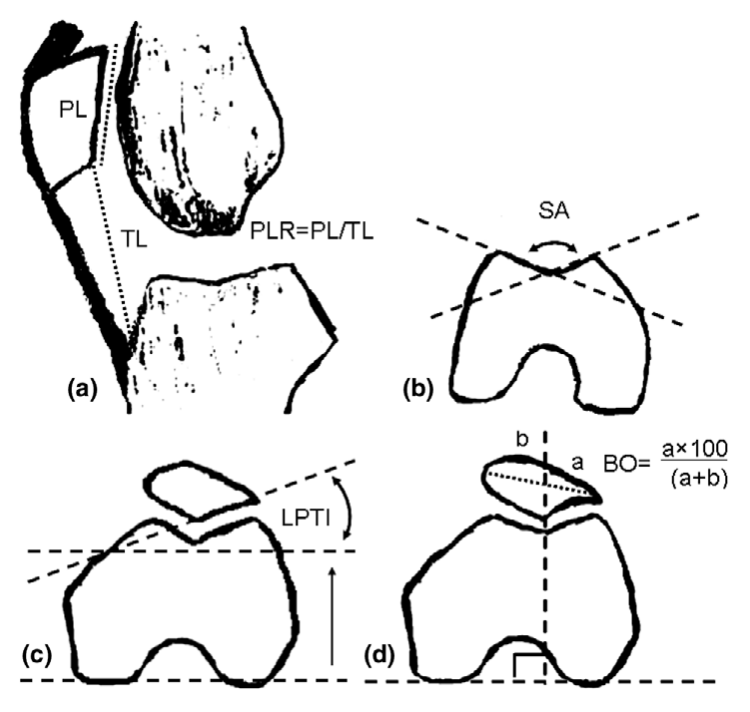
Diagram of measured patellar alignment indices. **(a) **In the sagittal plane, PL is the inner patellar length and TL is the patellar tendon length (PLR, the patellar length ratio, was computed as PL/TL). **(b-d) **In a transverse (axial) plane, SA is the sulcus angle **(b) **and LPTA is the lateral patellar tilt angle **(c)**; **(d) **diagram of bisect offset (BO) measurement.

In the present study we evaluated MRIs that had previously been acquired for BOKS. The patellar alignment evaluation for MRI in this study was performed with eFilm Workstation (version 2.0.0) software. We measured patellar alignment in two planes: sagittal and transverse (axial). In the sagittal plane we measured the patellar length ratio (PLR) by the Insall and Salvati method [13]. For these measurements we found the slice with clearly recognizable patellar margins and where the patellar bone volume seemed to be maximal. To measure patellar length and patellar ligament length by the Insall and Salvati method we constructed two lines (Figure 1a): patellar length, from the upper to the lower point of the inner (articulating) surface of the patella excluding osteophytes amd patellar ligament length, from the lower inner point of the patella to the highest point of tibial tuberosity. PLR was calculated as (Patellar length)/(Patellar ligament length).

In the transverse (axial) plane we measured two groups of indices: first, the index that describes the trochlear depth, namely SA [6,27,28], and second, indices that describe patellar position:, namely LPTA and BO [27,29,30]. For the measurements of SA we found the axial slice that referred to the proximal one-third of the femoral trochlear curve by using the three-dimensional cursor on the sagittal image. SA is the angle between two lines: from the lowest point of the trochlear sulcus, one on a lateral bony margin and the second on a medial bony margin (Figure 1b). For the measurements of patellar alignment we found the axial slice that refers to the middle of the patella by using the three-dimensional cursor on the sagittal image. LPTA is the angle between the posterior condylar line and a line drawn through the lateral interior bony margin of the patella (Figure 1c). For BO measurements we drew the posterior condylar line and perpendicular line up though the lowest point of the femoral sulcus and through the patella, and measured the distance between the lateral border of the patella and this vertical line (*a*) and between the medial border of the patella and this vertical line (*b*) (Figure 1d). BO was calculated from the formula BO = 100*a*/(*a *+ *b*).

### Reliability of MRI readings

First, we (LK and DH) read a batch of MRIs and decided on an exact protocol of evaluation of patellar alignment. Using this protocol, 10 MRIs were read and re-read by these two investigators separately to estimate the intra-rater and inter-rater reliability of the readings of each of the patellar alignment features. One investigator (LK) read the remainder of the MRIs, blinded to patient identifiers. To evaluate for reader drift, we re-assessed intra-rater reliability by inserting one original reliability scan for every 10 new scans. Before reading each batch of MRIs, LK re-read five previously read MRIs to 'calibrate' his readings against a standard. The intra-observer reliability intraclass correlation coefficient for reading for different patellar alignment indices varied between 0.86 and 0.96.

### Radiographic evaluation of PF OA

Patients underwent weight-bearing skyline radiography with the protocol of Buckland-Wright [31]. Theskyline viewradiographs were read by an academically based rheumatologist (DTF). The presence of osteophytes in the medial and lateral parts of the patella and femur as well as joint space narrowing in themedial and lateral parts of the PF joint were each graded on a four-point scale (range 0 to 3).

### Statistical analysis

The goal of our analysis was to evaluate the association between MRI measures of alignment and radiographic PF OA. We first categorized each of the four patellar alignment measurements into quartiles. Medial PF osteophytes took on whole-number values from 0 to 3, and were analyzed as ordered categories. We examined the relation between quartiles of each patellar alignment measure and medial PF osteophytes with the use of the proportional odds logistic regression model. A generalized estimating equation correction was applied to account for the correlation in the osteophytes outcome between the femur and patella within a knee. We then tested for linear trend between patellar alignment evaluation and medial PF cartilage by using patellar alignment evaluation as a continuous variable in the model. If there was potential U-shapes or J-shaped relation between a patellar alignment evaluation and medial PF cartilage, we tested the U-shaped trend by including both patellar alignment evaluation and its square. We used the same approach to examine the relation between each patellar alignment measure and lateral PF osteophytes. All models were adjusted for age, sex, and bone mass index (BMI). We also examined the relation between quartiles of each patellar alignment evaluation and medial PF joint space narrowing with the use of the proportional odds logistic regression model while adjusting for age, sex, and BMI. We then tested for linear trend and U-shaped trend. The same approach was used to examine the relation between each patellar alignment evaluation and lateral PF joint space narrowing. Statistical analyses were performed with SAS software (release 9.1; SAS Institute Inc, Cary, NC, USA).

## Results

Of the 324 patients entering BOKS, 311 obtained an MRI of their more symptomatic knee at baseline. Table 1 shows the characteristics of the 213 study participants selected at random from the larger study sample. We compared the group of individuals who were included in the present study (*n *= 213) with the group of individuals who were not (*n *= 111). There were no statistically significant differences between groups in terms of age (66.6 ± 9.3 versus 67.8 ± 9.1 respectively, *p *= 0.28) and BMI (31.4 ± 5.5 versus 31.5 ± 6.1 respectively, *p *= 0.87). This study sample was composed of 126 males (average age 68.0 years) and 87 females (average age 64.7 years). On average, the subjects were obese, with a mean BMI of 31.2 for males and 31.6 for females, and had radiographic knee OA (a Kellgren-Lawrence score of 2 or more in 65.9% of males and 87.4% of females).

**Table 1:** Characteristics of the study sample

Characteristics	*n*	Mean	Frequency (percentage)	Range
Age (years)	213	66.6	-	47–93
Sex (women)	213	-	40.8	-
Bone mass index	213	31.4	-	21.5–55.9
K-L ≥ 2	212	-	75.0	0–4

K-L, Kellgren-Lawrence score.

Tables 2 to 5 show the relation between patellar alignment measures and radiographic indices of PF OA. Each table presents the number of measured knees in each quartile, the range of patellar alignment measures in each quartile, odds ratios and the *p *for trend of the model.

**Table 2:** Association between patellar alignment (fore groups) and adjusted means of lateral PF joint space narrowing

Measure		Lateral joint space narrowing				*p *for trend
			
	Quartile...	1	2	3	4	
PLR	No. of knees	50	50	50	52	
	Range of PLR	0.66–0.87	0.88–0.98	0.98–1.12	1.13–1.71	
	OR (95% CI)	1.00	1.56 (0.66–3.67)	1.36 (0.57–3.23)	2.77 (1.20–6.39)	Linear, 0.0136; U-shaped, 0.1630
SA	No. of knees	51	52	49	50	
	Range of SA	98–113	114–119	120–124	125–155	
	OR (95% CI)	1.00	1.48 (0.66–3.33)	1.58 (0.71–3.56)	1.43 (0.63–3.24)	Linear, 0.1206; U-shaped, 0.6204
LPTA	No. of knees	52	51	44	54	
	Range of LPTA	-25 to 13	14–17	18–21	22–35	
	OR (95% CI)	1.00	0.46 (0.21–0.97)	0.32 (0.14–0.73)	0.10 (0.04–0.27)	Linear, <0.0001; U-shaped, 0.9073
BO	No. of knees	49	49	51	50	
	Range of BO	38.46–54.55	54.76–60.42	60.47–66.67	66.67–100	
	OR (95% CI)	1.00	2.16 (0.78–5.96)	4.22 (1.58–11.25)	8.26 (3.06–22.30)	Linear, <0.0001; U-shaped, 0.2468

Results are adjusted for age, sex and bone mass index. PLR, patellar length ratio; SA, sulcus angle; LPTA, lateral patellar tilt angle; BO, bisect offset; OR, odds ratio; CI, confidence interval.

**Table 3:** Association between patella alignment (fore groups) and adjusted means of medial PF joint space narrowing

Measure		Medial joint space narrowing				*p *for trend
			
	Quartile...	1	2	3	4	
PLR	No. of knees	50	50	50	52	
	Range of PLR	0.66–0.87	0.88–0.98	0.98–1.12	1.13–1.71	
	OR (95% CI)	1.00	1.97 (0.65–5.99)	2.09 (0.70–6.19)	2.47 (0.86–7.14)	Linear, 0.1253
SA	No. of knees	51	52	49	50	
	Range of SA	98–113	114–119	120–124	125–155	
	OR (95% CI)	1.00	1.37 (0.47–3.98)	1.66 (0.57–4.87)	3.17 (1.15–8.72)	Linear, 0.0162
LPTA	No. of knees	52	52	44	54	
	Range of LPTA	-25 to 13	14–17	18–21	22–35	
	OR (95% CI)	1.00	1.532 (0.546–4.302)	1.697 (0.603–4.773)	2.185 (0.822–5.809)	Linear, 0.0259
BO	No. of knees	49	49	51	50	
	Range of BO	38.46–54.55	54.76–60.42	60.47–66.67	66.67–100	
	OR (95% CI)	1.00	0.887 (0.346–2.272)	0.711 (0.272–1.857)	0.189 (0.057–0.638)	Linear, 0.0026

Results are adjusted for age, sex and bone mass index. PLR, patellar length ratio; SA, sulcus angle; LPTA, lateral patellar tilt angle; BO, bisect offset; OR, odds ratio; CI, confidence interval.

**Table 4:** Association between patella alignment (fore groups) and adjusted means of lateral patellar osteophytes

Measure		Lateral patellar osteophytes				*p *for trend
			
	Quartile...	1	2	3	4	
PLR	No. of knees	100	100	100	104	
	Range of PLR	0.66–0.87	0.88–0.98	0.98–1.12	1.13–1.71	
	OR (95% CI)	1.00	1.70 (1.01–2.86)	1.23 (0.73–2.08)	1.67 (0.98–2.84)	Linear, 0.0138; U-shaped, 0.0943
SA	No. of knees	102	104	98	100	
	Range of SA	98–113	114–119	120–124	125–155	
	OR (95% CI)	1.00	1.62 (0.97–2.71)	1.83 (1.09–3.08)	1.52 (0.91–2.55)	Linear, 0.0804; U-shaped, 0.8875
LPTA	No. of knees	104	102	88	108	
	Range of LPTA	-25 to 13	14–17	18–21	22–35	
	OR (95% CI)	1.00	0.35 (0.21–0.60)	0.51 (0.30–0.88)	0.29 (0.17–0.49)	Linear, <0.0001; U-shaped, 0.1076
BO	No. of knees	98	98	102	100	
	Range of BO	38.46–54.55	54.76–60.42	60.47–66.67	66.67–100	
	OR (95% CI)	1.00	0.92 (0.54–1.54)	1.33 (0.79–2.25)	3.07 (1.77–5.34)	Linear, <0.0001; U-shaped, 0.2038

Results are adjusted for age, sex and bone mass index. PLR, patellar length ratio; SA, sulcus angle; LPTA, lateral patellar tilt angle; BO, bisect offset; OR, odds ratio; CI, confidence interval.

**Table 5:** Association between patella alignment (fore groups) and adjusted means of medial patellar osteophytes

Measure		Medial patellar osteophytes				*p *for trend
			
	Quartile...	1	2	3	4	
PLR	No. of knees	100	100	100	104	
	Range of PLR	0.66–0.87	0.88–0.98	0.98–1.12	1.13–1.71	
	OR (95% CI)	1.00	1.27 (0.75–2.15)	1.16 (0.68–1.96)	1.12 (0.66–1.90)	Linear, 0.5996
SA	No. of knees	102	104	98	100	
	Range of SA	98–113	114–119	120–124	125–155	
	OR (95% CI)	1.00	1.465 (0.87–2.43)	1.73 (1.03–2.93)	1.69 (1.01–2.83)	Linear, 0.0514
LPTA	No. of knees	104	102	88	108	
	Range of LPTA	-25 to 13	14–17	18–21	22–35	
	OR (95% CI)	1.00	0.80 (0.48–1.35)	0.93 (0.55–1.60)	1.43 (0.85–2.39)	Linear, 0.1080
BO	No. of knees	98	98	102	100	
	Range of BO	38.46–54.55	54.76–60.42	60.47–66.67	66.67–100	
	OR (95% CI)	1.00	0.887 (0.346–2.272)	0.711 (0.272–1.857)	0.189 (0.057–0.638)	Linear, 0.888

Results are adjusted for age, sex and bone mass index. PLR, patellar length ratio; SA, sulcus angle; LPTA, lateral patellar tilt angle; BO, bisect offset; OR, odds ratio; CI, confidence interval.

PLR showed a statistically significant association with individual radiographic features, namely osteophytes and joint space narrowing of PF OA in the lateral compartment. The lowest frequency of lateral joint space narrowing was found in PLR ranges 0.66 to 0.87 (lowest PLR, referent quartile). With increasing PLR there was an increased risk of lateral joint space narrowing; odds ratios for quartiles were 1.00 (lowest PLR, referent quartile), 1.56, 1.36, and 2.77 (highest quartile) (*p *for linear trend = 0.01). A similar trend was found between increasing PLR and increasing lateral patellar osteophytosis; odds ratios were 1.00, 1.70, 1.23, and 1.67 (*p *for linear trend = 0.01). There was no statistically significant association between PLR and indices of radiographic PF OA in the medial PF compartment.

SA showed a statistically significant association with medial joint space narrowing and lateral and medial patellar osteophytosis. With increasing SA there was increased risk of medial joint space narrowing; odds ratios were 1.00 (referent quartile, SA range 98 to 113°), 1.37, 1.66, and 3.16 (the highest quartile, SA range 125 to 155°) (*p *for linear trend = 0.01). For lateral patellar osteophytosis the odds ratios were 1,.00 1.62, 1.83, and 1.52 (*p *for linear trend = 0.08). For medial patellar osteophytosis the odds ratios were 1.00, 1.45, 1.73, and 1.69 (*p *for linear trend = 0.05).

LPTA showed a statistically significant association with joint space narrowing and osteophytosis of the lateral PF compartment. The lowest range (referent quartile) of LPTA values spanning -25 to 13° was associated with the greatest lateral joint space narrowing; odds ratios were 1.00, 0.46, 0.32, and 0.10 (*p *for linear trend < 0.0001). A similar association was found between LPTA and lateral patellar osteophytosis, with odds ratios being 1.00, 0.35, 0.51, and 0.29, respectively (*p *for linear trend < 0.0001).

BO showed statistically significant associations with lateral and medial joint space narrowing and lateral PF osteophytosis. A more laterally displaced patella was associated with increased lateral joint space narrowing; odds ratios were 1.00, 2.16, 4.22, and 8.26 (*p *for linear trend < 0.0001). It was also positively associated with lateral patellar osteophytosis; odds ratios were 100, 0.92, 1.33, and 3.07 (*p *for linear trend < 0.0001). However, laterally displaced patella was negatively associated with medial joint space narrowing; odds ratios were 1.00, 0.89, 0.71, and 0.19 (*p *for linear trend < 0.0026). Thus, increasing medial displacement of the patella was associated with medial joint space narrowing.

## Discussion

In the present cross-sectional study we found significant associations between patellar alignment evaluated with standard knee MRI and indices of radiographic PF OA, such as joint space narrowing and patellar osteophytes.

PLR is a measure of the vertical position of the patella measured on the lateral view and was originally proposed by Insall and Salvati [13]. Shabshin and colleagues [32] used MRIs of extended knees to measure the PLR, and suggested that PLRs of more than 1.50 or less than 0.74 define patella alta and patella baja, respectively. Previous studies suggested that a high-riding patella (patella alta) can be associated with lateral patellar dislocation and subluxation, chondromalacia patellae, patellar ligament rupture, and Sinding-Larsen-Johansson disease, patellar and quadriceps tendonitis, and Osgood-Schlatter disease [13,14,28,33-36]. Our study demonstrated that increasing PLR is significantly associated with increasing joint space narrowing and osteophytoses in the lateral compartment of the PF joint. These results are similar to previously published data [35] investigating the close association of idiopathic retropatellar pain with patella alta.

The patella increases the mechanical advantage of extensor muscles by transmitting forces across the knee at a greater distance (moment) from the axis of rotation, thus increasing the functional lever arm of the quadriceps as well as changing the direction of pull of the quadriceps mechanism. A longer patellar tendon decreases the patellar advantage as a functional lever arm of the quadriceps in commonly used angles of knee flexion (30 to 60°), which can increase compression in the PF joint and can therefore lead to excessive cartilage attrition and joint space narrowing. Another potential explanation is that a high-riding patella placed against a much shallower femoral sulcus can lead to instability (the evidence for this is that a higher rate of patellar dislocation is associated with patella alta) that can lead to the degeneration of cartilage and osteophyte formation.

In our study SA was significantly associated with lateral and medial patellar osteophytosis and also with medial joint space narrowing. SA is an indicator of femoral trochlear dysplasia, one of the anomalies associated with PF OA and with patellar instability [37]. Patellar instability can cause excessive traction and compression forces on both sides of the patella itself and both the patellar and femoral articular cartilages, and can potentially aggravate osteophyte formation and also facilitate joint space narrowing.

In the present study we used two indices of PF relationship: LPTA and BO. Both measures showed statistically significant positive associations with PF OA in the lateral compartment. BO demonstrated a negative association with medial joint space narrowing. There are several possible explanations for our findings, although it should be recognized that this is a cross-sectional study and any causal inference is not possible with such a design. BO indicates the lateral displacement of the patella in relation to deepest part of the femoral sulcus. LPTA shows the angle of patellar inclination, which indicates the tightness or looseness of the lateral stabilizing mechanism of the patella. MRIs in our study were taken in a supine position and with fully extended knees with the quadriceps relaxed. If we found a laterally displaced patella and/or lateral border of patella too close to the lateral femoral condyle (decreased LPTA) on those images it could mean that the structures that hold it in the lateral position (lateral retinaculum, vastus lateralis) were shortened. In this situation, during knee movement the patella would be compressed against the lateral femoral condyle, rather than distributing load evenly between the lateral and medial PF compartments. Excessive compressive forces primarily located on the lateral PF compartment in combination with movement could lead to wear on the cartilage and, as a result, to its degeneration. An alternative explanation could be that OA changes caused the alteration in patellar alignment; that is, with increased narrowing of the radiographic joint space in the lateral compartment this allowed lateral displacement of the patella with reference to the femur.

There were numerous limitations of the present study that need to be recognized. First, the MRI images were performed in a supine position rather than a weight-bearing one. This limitation is likely to have reduced our opportunity to measure dynamic changes in patella position with weight bearing and thus underscore the fact that our findings are likely to be conservative for measures that could potentially change with weight bearing such as BO and the LPTA. Second, the MRI was obtained in a fully extended knee. This position, as mentioned above, is common in clinical practice, but in the extended knee the patella is not positioned against the trochlear sulcus and it makes the measurement of their congruence less precise. Third, our study was cross-sectional, and any evidence of causality needs to be explored further in longitudinal studies. Although the study may be internally valid it is not necessarily generalizable to other persons with symptomatic knee OA.

## Conclusion

A full understanding of the risk factors for OA in the PF joint requires the consideration of a range of different risk factors. The alignment of the patella may be an important factor influencing PF joint degeneration due to the aberrant distribution of forces with activity. On the basis of the results of this study it does seem that non-weight-bearing, full-extension assessment of patellar alignment does increase our understanding of the reasons for PF OA. The results of our study suggest that indices of patellar alignment can be measured easily on a standard knee MRI. Statistically significant associations were found between indices of patellar alignment and such features of PF OA as osteophytosis and joint space narrowing. Further consideration needs to be given to the importance of PF alignment, preferably in more functional positions than supine and non-weight-bearing, and in longitudinal evaluations.

## Abbreviations

BMI = bone mass index; BO = bisect offset; BOKS = Boston Osteoarthritis of the Knee Study; LPTA = lateral patellar tilt angle; MRI = magnetic resonance imaging; OA = osteoarthritis; PF = patello-femoral; PLR = patellar length ratio; SA = sulcus angle.

## Competing interests

The authors declare that they have no competing interests.

## Authors' contributions

LK participated in the design and coordination of the study, read the MRIs, and prepared the manuscript. YZ participated in the design of the study. JN performed the statistical analyses. JG participated in the sequence alignment. DG read MRIs. DTF read the X-rays and made substantial contributions to the design concept. DJH conceived of the study, participated in the design and coordination of the study, and helped to draft the manuscript. All authors read and approved the final manuscript.
